# Lack of Evidence for Seasonal Variation in Herpes Zoster: A Nine-Year Retrospective Cross-Sectional Study

**DOI:** 10.3390/microorganisms14102392

**Published:** 2026-10-19

**Authors:** Zuhal Erçin, Mehtap Toprak

**Affiliations:** 1 Department of Dermatology, Sultan 2. Abdulhamid Han Training and Research Hospital, University of Health Sciences, 34668 Istanbul, Turkey; 2 Department of Pathology, Sultan 2. Abdulhamid Han Training and Research Hospital, University of Health Sciences, 34668 Istanbul, Turkey; dr.mehtapguner@hotmail.com

**Keywords:** herpes zoster, seasonality, epidemiology

## Abstract

Herpes zoster results from the reactivation of latent varicella-zoster virus and may cause serious complications. Although traditionally considered nonseasonal, recent studies have reported summer peaks in herpes zoster occurrence. Given these inconsistencies, monthly and seasonal variations in herpes zoster case rates and their implications for prevention were evaluated in this study. In this retrospective cross-sectional study, the medical records of patients diagnosed with herpes zoster at a dermatology outpatient clinic between January 2017 and December 2025 were reviewed. Data on age, sex, and the year, month, and season of diagnosis were collected and analyzed using IBM SPSS Statistics for Windows, version 30.0. The highest proportion of herpes zoster cases occurred in patients aged 60–69 years. Visit-adjusted case rates did not differ significantly across months or seasons. A significant increasing trend in herpes zoster case rates was observed from 2020 to 2025. Herpes zoster is a vaccine-preventable disease that may cause serious complications and shows an increasing trend in occurrence. No statistically significant seasonal variation in herpes zoster occurrence was detected in this cohort, supporting the importance of maintaining year-round preventive strategies, including vaccination.

## 1. Introduction

Varicella-zoster virus (VZV), also known as human herpesvirus 3 (HHV-3), is a double-stranded DNA alphaherpesvirus that causes varicella (chickenpox) during primary infection and herpes zoster (shingles) following the reactivation of latent virus. Primary VZV infection generally occurs during childhood and is transmitted through respiratory droplets or direct contact with infectious secretions [1]. After initial infection, VZV spreads to the dorsal root or trigeminal nerve ganglia via blood or retrogradely from cutaneous nerves and remains latent there for life [2]. The development of herpes zoster is triggered by the reactivation of latent VZV acquired through primary infection or vaccination, usually in the setting of waning immunity [3]. Cell-mediated immunity plays a central role in maintaining VZV latency, and reactivation is generally attributed to a decline in this immune surveillance. Upon reactivation, VZV replicates within cell bodies of neurons [4]. In the subsequent phase, the viral particles are transported from the cell bodies along the nerve axons to the associated dermatome. In the affected dermatome, the virus provokes inflammation and causes a painful, erythematous, vesicular rash [1]. VZV shows a worldwide distribution. Varicella infection in pregnancy can spread via the placenta, leading to fetal infection, which leads to disseminated life-threatening diseases. However, herpes zoster does not pose a risk to a developing fetus due to specific maternal antibodies that are transmitted diaplacental to the fetus [4]. The characteristic cutaneous manifestation of herpes zoster is a painful, grouped vesicular rash that is usually limited to a single dermatomal distribution [5]. In immunocompromised individuals, herpes zoster may present with disseminated disease involving multiple organs, including the brain and liver [2,5]. Herpes zoster may lead to severe complications, resulting in increased hospitalization rates, but it is uncommon in the pediatric population [5]. Notable complications of herpes zoster include postherpetic neuralgia, cranial nerve palsies, meningitis, encephalitis, myelitis, stroke, bacterial superinfections, hepatitis, Reye syndrome, and Ramsay Hunt syndrome [1,5]. Although postherpetic neuralgia is the most common neurological complication of herpes zoster, stroke represents one of its most serious complications. A total of 10–20% of patients with herpes zoster suffer from herpes zoster ophthalmicus, the infection of the ophthalmic branch of the trigeminal nerve, which can cause vision loss. The risk of stroke increases after a herpes zoster episode, especially following cases with ocular involvement and in immunocompromised patients. Well-established risk factors for herpes zoster include advanced age (≥50 years), immunodeficiency, acute infections, psychological stress, and female sex [1]. Herpes zoster has traditionally been considered to occur throughout the year without epidemic patterns, reflecting the reactivation of latent endogenous VZV, with most population-based studies reporting no seasonal variation in incidence [6,7]. Herpes zoster can occur at any age, particularly in individuals with impaired cell-mediated immunity due to underlying diseases or immunosuppressive medications [8]. Recent large population-based studies have demonstrated significant seasonal variation with a peak during the summer months [9,10]. To address the inconsistent findings of previous studies on herpes zoster seasonality, this study aimed to evaluate monthly and seasonal variation and temporal trends of herpes zoster and to investigate their potential implications for disease prevention.

## 2. Materials and Methods

This study was conducted in accordance with the Declaration of Helsinki, and the protocol was approved by the Ethics Committee of Istanbul Medipol University with protocol code E-10840098-202.3.02-3570 on 4 June 2025. As anonymized data from routinely collected outpatient clinical records were analyzed in this retrospective study, informed consent for participation was not required in accordance with the Turkish Regulation on Clinical Research, overseen by the Turkish Medicines and Medical Devices Agency, Ministry of Health. The clinical photographs contain no identifiable information, and informed consent for their publication was obtained from the patients or their legal guardians, as applicable. A retrospective cross-sectional study design was used, wherein the outpatient records of all patients who visited the dermatology outpatient clinic of our hospital between January 2017 and December 2025 were reviewed. Outpatient clinic records were screened for herpes zoster using the ICD-10 (International Classification of Diseases, 10th Revision) code B02. Diagnoses identified using ICD-10 code B02 were verified through a review of clinical records. Patients were identified using their names and unique hospital identification numbers. To avoid duplicate counting, each patient was included only once throughout the study period, and subsequent records associated with the same patient were excluded from the analysis. Patient-identifying information was removed from the dataset prior to statistical analysis. Medical records of all patients diagnosed with herpes zoster, regardless of age, were retrospectively reviewed. Data on patient age, sex, and the year, month, and season of herpes zoster diagnosis were collected. Because the study was conducted in the Northern Hemisphere, December, January, and February were classified as winter; March, April, and May as spring; June, July, and August as summer; and September, October, and November as autumn. The data obtained from the study were analyzed using IBM SPSS Statistics for Windows, version 30.0 (IBM Corp., Armonk, NY, USA). Descriptive statistics for the sociodemographic and clinical characteristics of the patients were presented as counts (*n*), percentages (%), medians, and interquartile ranges (IQR). The distributions of herpes zoster cases according to month, season, and year were presented using frequency tables and graphs. Monthly and annual pooled herpes zoster case rates were calculated by dividing the total number of herpes zoster cases by the total number of dermatology outpatient visits during the corresponding periods and multiplying the result by 1000. Differences in herpes zoster case proportions across months, seasons, and years were evaluated using the chi-square test. When a significant difference was detected, pairwise comparisons of proportions were performed with Bonferroni adjustment for multiple comparisons. Additionally, negative binomial regression was performed to assess differences in monthly herpes zoster case rates while adjusting for calendar year. Monthly herpes zoster case counts were used as the dependent variable, with calendar month and year included as categorical independent variables. The natural logarithm of the monthly number of dermatology outpatient visits was included as an offset. The overall effect of calendar month was evaluated using a Wald chi-square test. A linear-by-linear association test was used to assess the presence of a linear trend across years. To evaluate more recent temporal trends, this analysis was specifically applied to the 2020–2025 period. Statistical significance was set at *p* < 0.05.

## 3. Results

**Figure 1:**
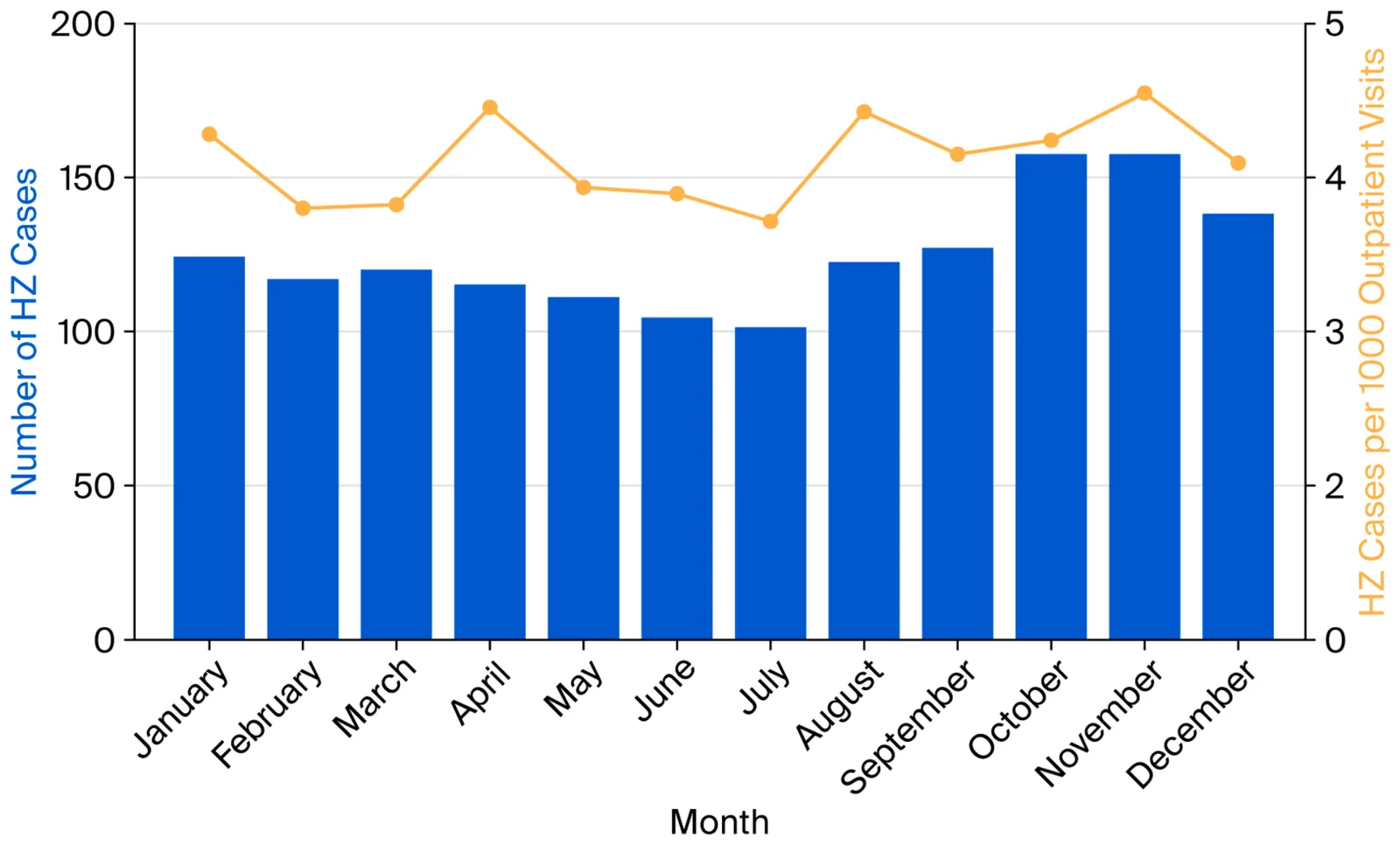
Total number of herpes zoster (HZ) cases and case rates per 1000 dermatology outpatient visits by month.

**Figure 2:**
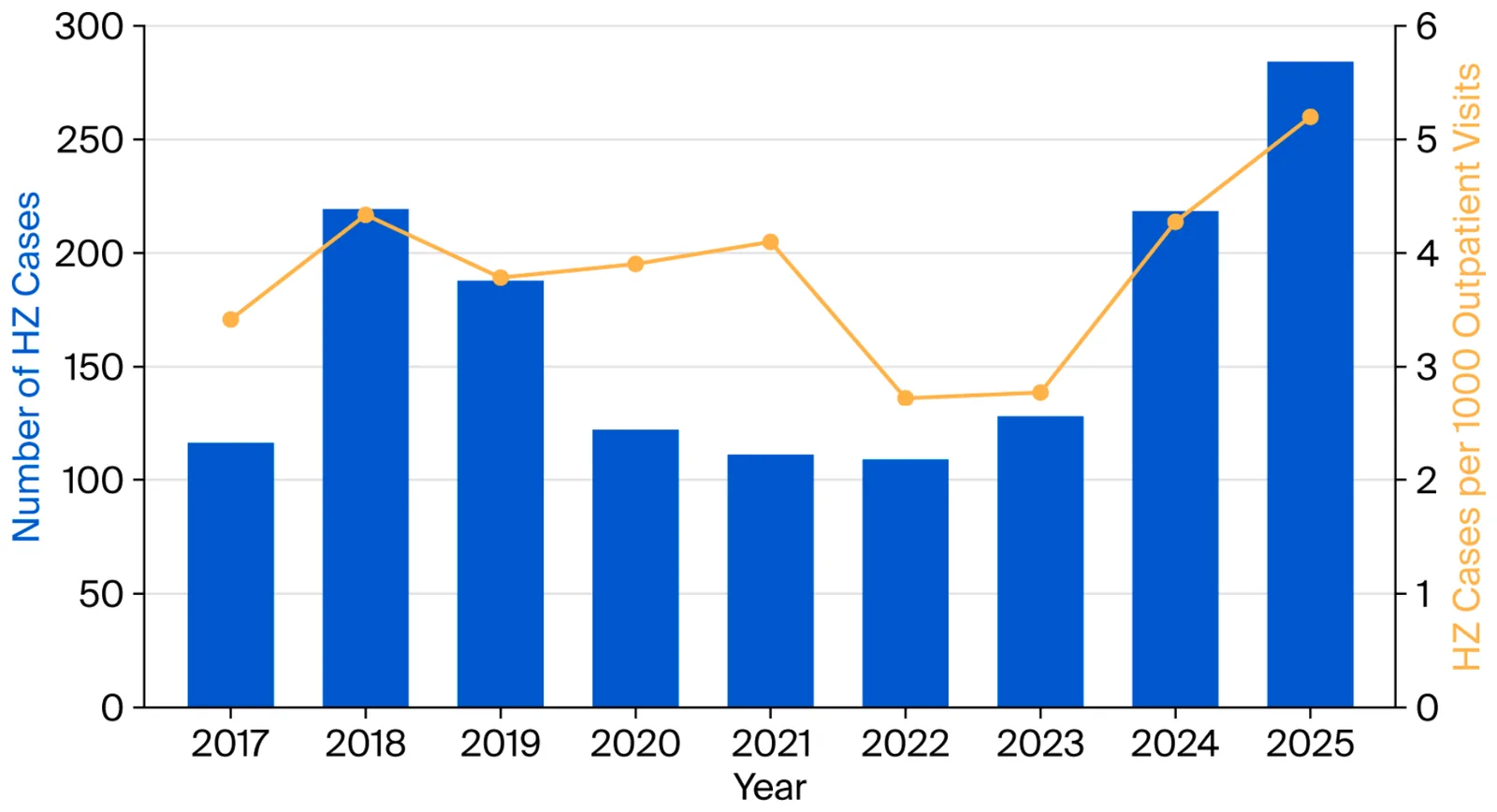
Total number of HZ cases and case rates per 1000 dermatology outpatient visits by year.

**Figure 3:**
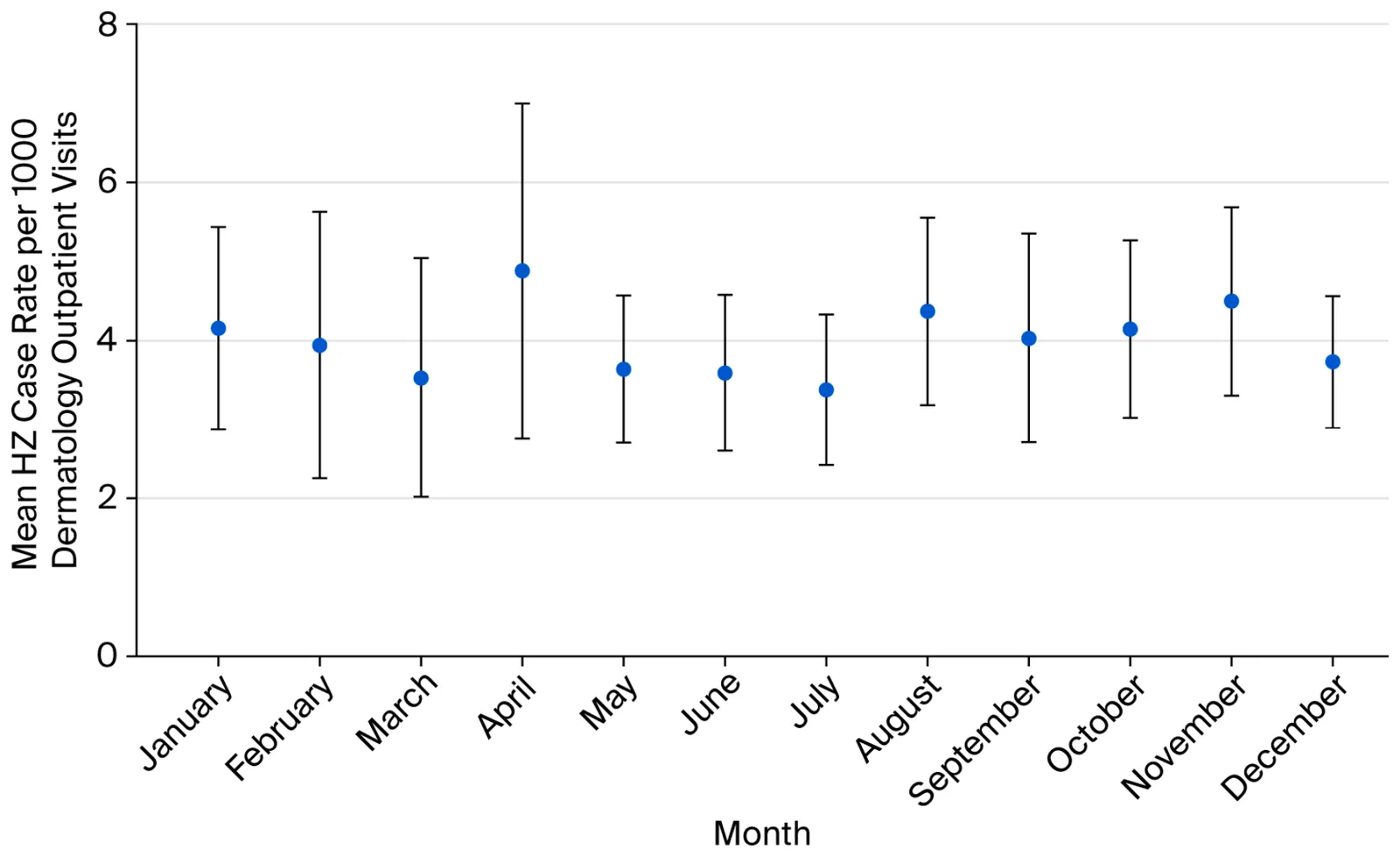
Mean monthly herpes zoster case rates per 1000 dermatology outpatient visits from 2017 to 2025. Points represent the mean rate for each calendar month across the nine-year study period, and error bars indicate 95% confidence intervals.

**Figure 4:**
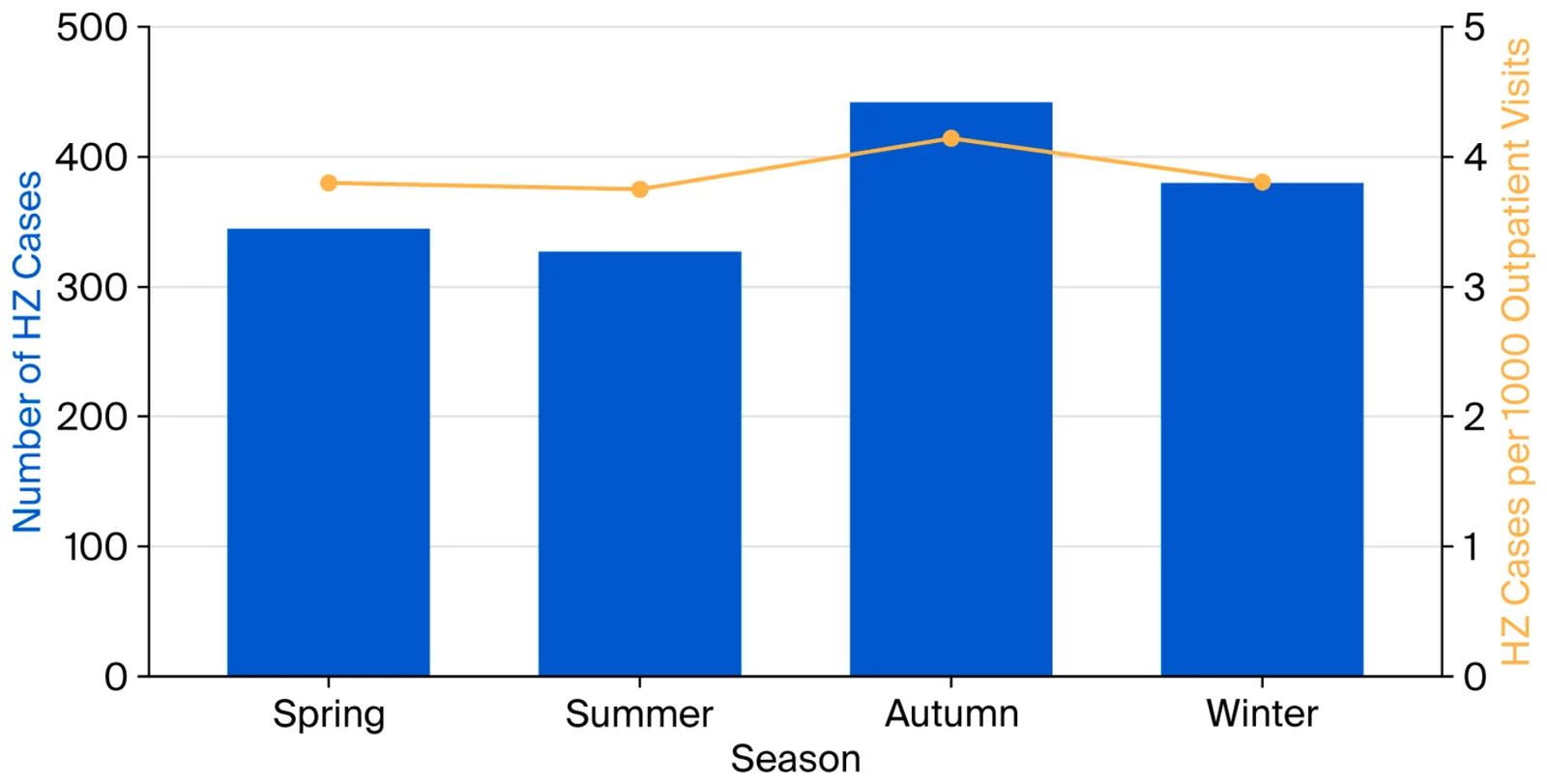
Total number of HZ cases and case rates per 1000 dermatology outpatient visits by season.

During the study period, a total of 1493 patients diagnosed with herpes zoster were evaluated; 784 (52.5%) were female and 709 (47.5%) were male. The patients ranged in age from 1 to 104 years, with a mean age of 53.4 ± 19.0 years and a median age of 56 years. The highest proportion of cases occurred in the 60–69-year age group (21.8%), followed by the 50–59-year age group (21.3%) (Table 1). The numbers of herpes zoster cases and total outpatient visits by year, month, and season are summarized in Table 2. The seasonal and annual variation in the herpes zoster case rate per 1000 dermatology outpatient visits is summarized in Table 3. The monthly and annual variation in the herpes zoster case rate per 1000 dermatology outpatient visits is summarized in Table 4. The overall pooled case-to-dermatology outpatient visit rate of herpes zoster was 3.89 cases per 1000 visits. The highest and second-highest monthly case rates were observed in April 2020 and April 2021, at 9.04 and 8.48 cases per 1000 visits, respectively. Monthly pooled rates ranged from 3.39 to 4.43 per 1000 visits, with the highest rate observed in November and the lowest rate in July (Figure 1). However, no statistically significant difference was observed in visit-adjusted case rates across months (*p* = 0.453) (Table 5). Annual pooled rates ranged between 2.72 and 5.21 cases per 1000 dermatology outpatient visits, with the highest rate observed in 2025 and the lowest rate in 2022 (Figure 2). Negative binomial regression analysis, adjusted for calendar year and incorporating the logarithm of the number of monthly dermatology outpatient visits as an offset, revealed no statistically significant differences in herpes zoster case rates across calendar months (Wald χ^2^(11) = 8.449, *p* = 0.673) (Figure 3). The study period began in January 2017, following our hospital’s transition from a military hospital to a public hospital in October 2016. No statistically significant linear trend in visit-adjusted herpes zoster case rates was observed over the entire study period (2017–2025; *p* = 0.051). However, a post hoc exploratory analysis restricted to 2020–2025 revealed a statistically significant increasing trend (*p* < 0.001). This restricted analysis was conducted to explore temporal changes during and following the COVID-19 pandemic. Bonferroni-adjusted post hoc analyses showed that the herpes zoster case rate in 2025 was significantly higher than those in 2017, 2018, 2019, 2022, and 2023, whereas the case rates in 2022 and 2023 were significantly lower than those in 2024. During the 2020–2025 period, a significant linear increasing trend in herpes zoster case rates was observed (Linear-by-Linear Association = 14.431; df (degrees of freedom) = 1; *p* < 0.001). However, this increase was not continuous over the years; herpes zoster case rates decreased in 2022 and 2023, followed by an increase in 2024 and 2025, reaching the highest rate in 2025 (5.21 per 1000 dermatology outpatient visits) (Table 6). In the seasonal analysis, the highest case rate was observed in autumn, with a rate of 4.14 per 1000 visits. The corresponding rates were 3.82 in winter, 3.80 in spring, and 3.76 in summer (Figure 4). However, the difference between seasons was not statistically significant (*p* = 0.486) (Table 7).

## 4. Discussion

**Figure 5:**
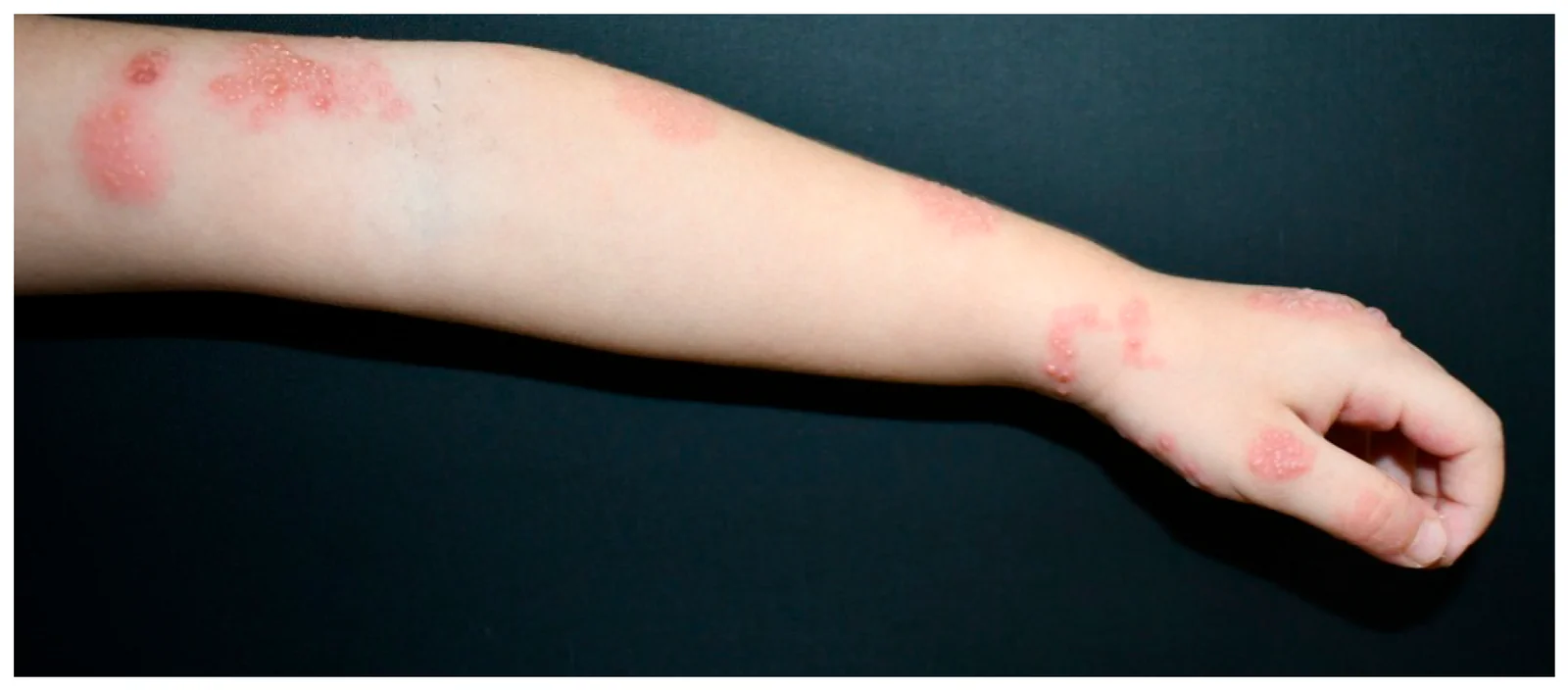
Herpes zoster lesions on the left arm of a 2-year-old girl.

**Figure 6:**
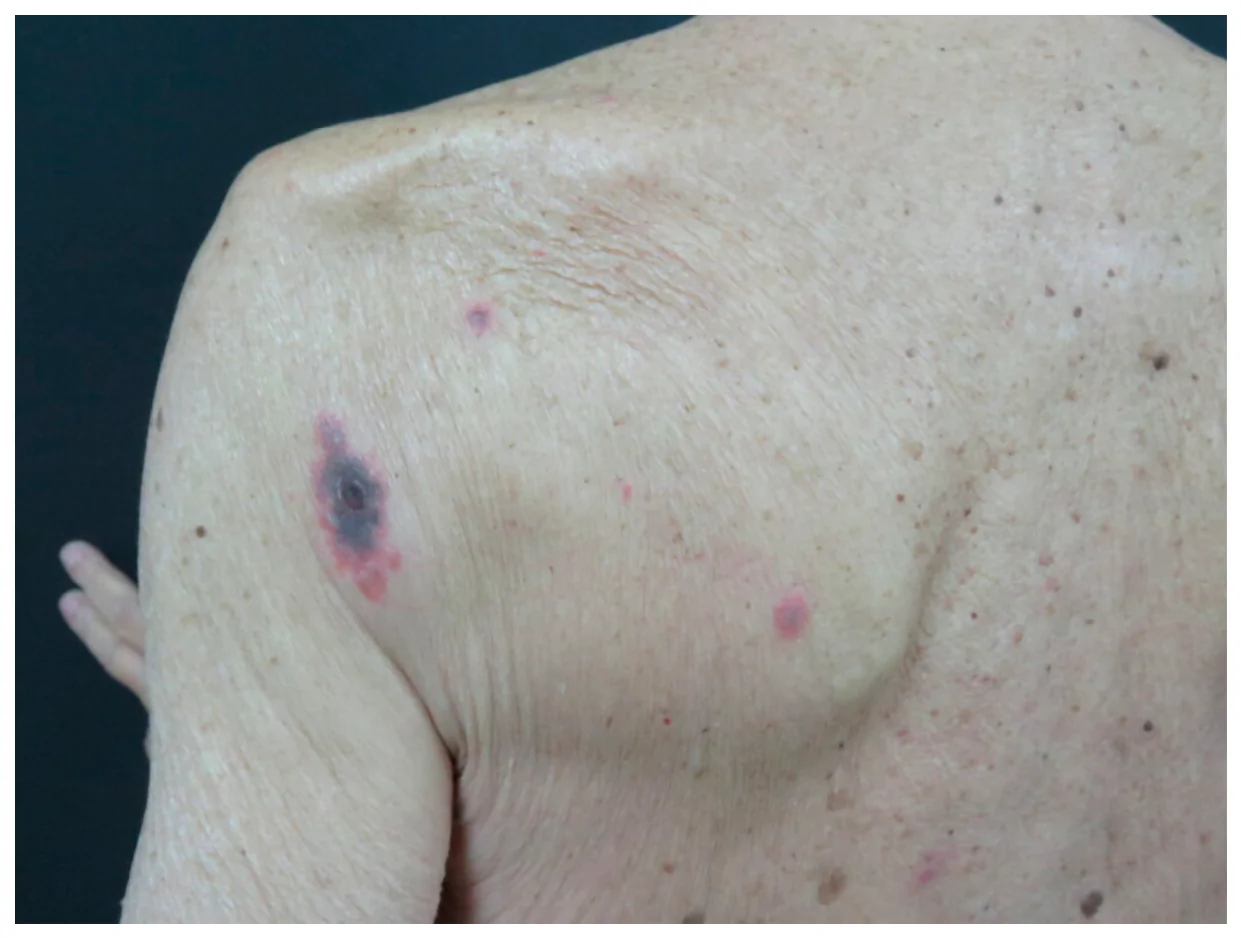
Disseminated herpes zoster in an 81-year-old patient with an atypical clinical presentation, confirmed by histopathological examination in the absence of available PCR testing.

**Figure 7:**
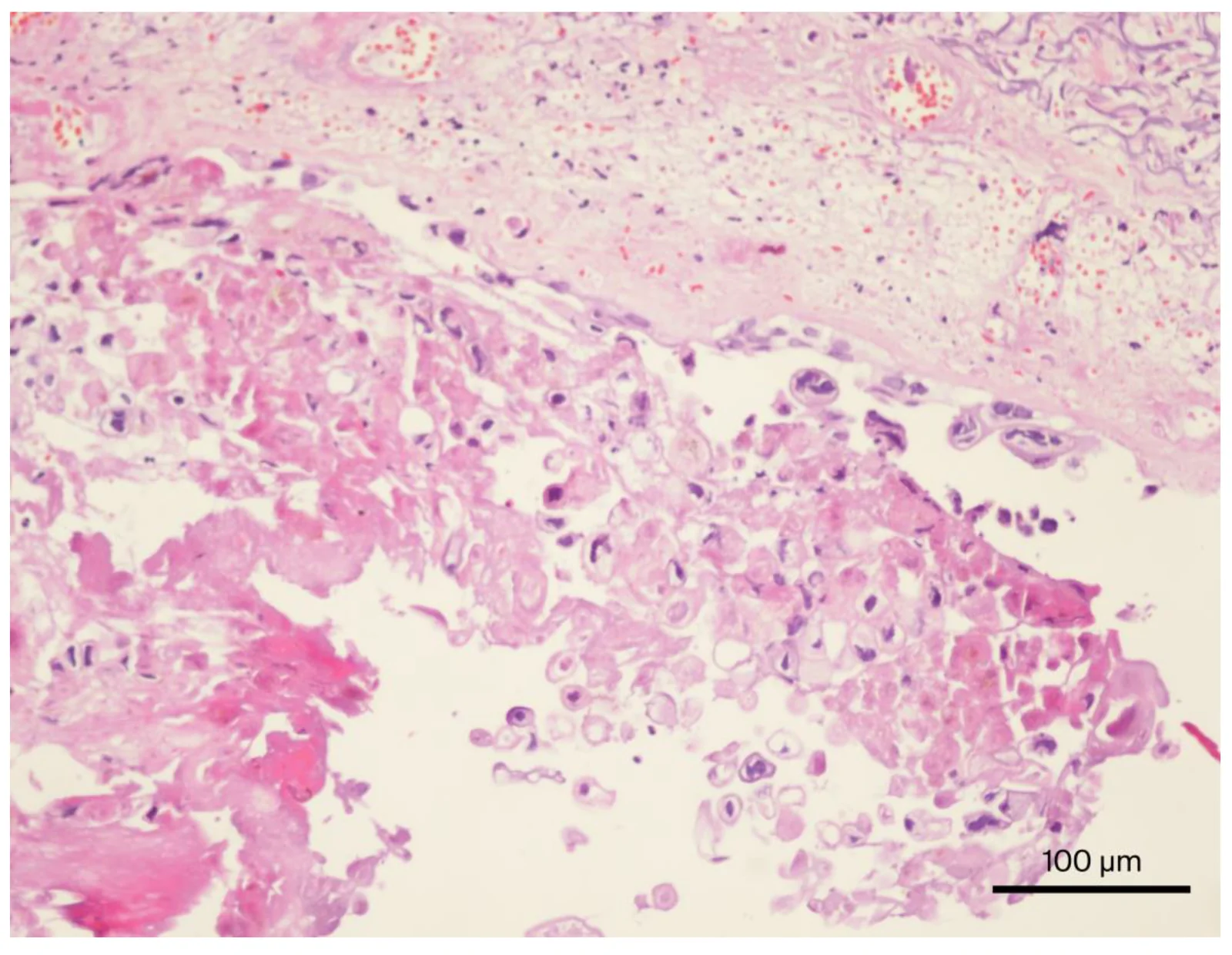
Histopathological examination of the patient shown in Figure 6, demonstrating multinucleated acantholytic keratinocytes—hematoxylin and eosin (H&E)-stained section (original magnification, ×20).

**Figure 8:**
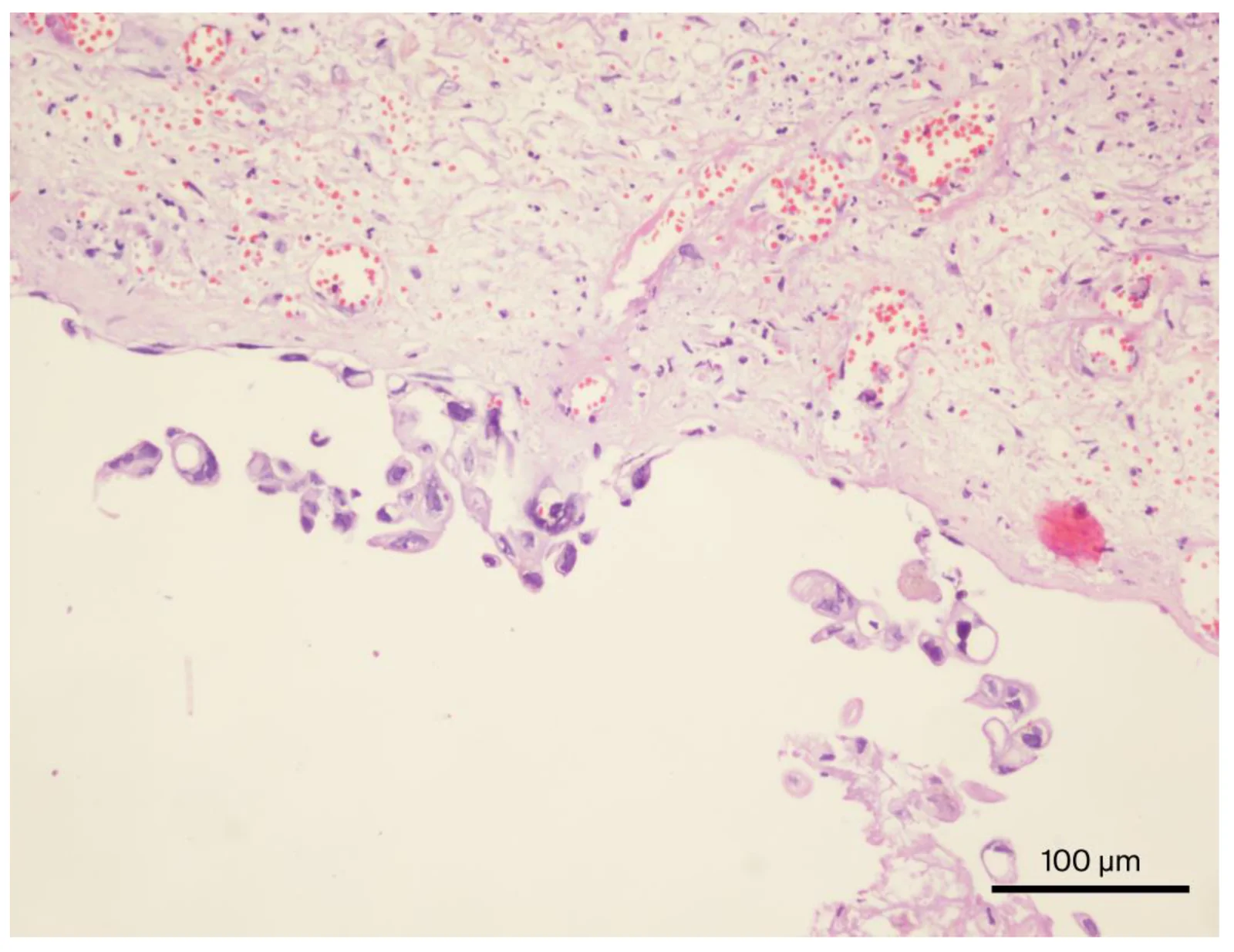
Histopathological examination of the patient shown in Figure 6, demonstrating ballooning degeneration and nuclear margination—H&E-stained section (original magnification, ×20).

Varicella-zoster virus is a highly contagious human herpesvirus that causes varicella during primary infection [1]. VZV can be transmitted through the airborne route or through direct contact with vesicular fluid from varicella or herpes zoster lesions [7]. Following primary infection, VZV establishes latency in sensory ganglia and may reactivate decades later, resulting in herpes zoster [1]. Upon reactivation, VZV is transported along microtubules within sensory axons to the skin, where it infects epithelial cells [7]. VZV reactivation is associated with impaired cell-mediated immunity, which may occur as part of the natural aging process, as a consequence of an immunocompromising condition, or following immunosuppressive therapy [7,8]. Herpes zoster occurs worldwide, and its complications are potentially life-threatening [4]. Evidence regarding the seasonality of herpes zoster remains inconsistent [9]. Given the inconsistent evidence regarding herpes zoster seasonality, this study aimed to assess monthly and seasonal variations in herpes zoster case rates and to explore their potential implications for disease prevention. A total of 1493 patients with herpes zoster were included in this study, of whom 52.5% were female and 47.5% were male, indicating a slight female predominance. This finding is consistent with the study conducted in Sweden, which provides evidence of the female predominance in herpes zoster disease [11]. The patients in our study ranged in age from 1 to 104 years, consistent with previous reports indicating that herpes zoster can occur across all age groups [8] (Figure 5 and Figure 6). The mean age of the patients was 53.4 ± 19.0 years, with a median age of 56 years. Adults above 50 years are at an increased risk for developing herpes zoster due to immunosenescence associated with advancing age [8]. Age-related immunosenescence is accompanied by a progressive decline in cell-mediated immunity, which may facilitate the reactivation of latent VZV [7]. The greatest proportion of our cases was observed in the 60–69-year age group (21.8%), followed closely by the 50–59-year age group (21.3%); together, these age groups accounted for 43.1% of all cases. This finding is consistent with a multicenter study conducted in our country, in which the largest proportion of herpes zoster cases was observed among individuals aged 60–69 years (24.3%), followed by those aged 50–59 years (20.3%), with these two age groups together accounting for 44.6% of all cases. Herpes zoster is not a notifiable disease in our country, and no nationwide surveillance or registry system is currently available [12]. Consequently, epidemiological data are primarily derived from single-center or multicenter studies rather than population-based studies. In our study, the highest and second-highest monthly case rates were observed in April 2020 and April 2021, coinciding with periods of stringent COVID-19-related public health restrictions in our country. The marked reduction in dermatology outpatient visits during these periods may have contributed to the elevated case rates per 1000 outpatient visits, potentially reflecting pandemic-related changes in healthcare utilization and healthcare-seeking behavior rather than a true increase in herpes zoster occurrence [13,14]. Monthly pooled herpes zoster case rates in our study ranged from 3.39 to 4.43 per 1000 dermatology outpatient visits, with the highest rate observed in November and the lowest in July; however, no statistically significant differences were observed across months. Previous population-based studies also reported no significant association between month and the occurrence of herpes zoster [15,16]. In the seasonal analysis, the highest herpes zoster case rate was observed in autumn, followed by winter, spring, and summer. However, these differences were not statistically significant, indicating no clear evidence of seasonal variation in herpes zoster case rates. A study conducted in 2025 similarly found no statistically significant seasonal variation in herpes zoster occurrence, although the mean number of cases was slightly higher during the summer months [17]. The multicenter study conducted in 2025 and a population-based study conducted in 2014 reported no apparent seasonal variation in herpes zoster occurrence; however, neither study performed a statistical analysis to formally assess seasonality [12,15]. In contrast to our findings, several studies have reported significant seasonal variation in herpes zoster occurrence, with a peak during the summer months [9,10,18]. A recent study from Germany identified two distinct peaks in herpes zoster incidence: one during the summer and another from late December to early January [19]. Two previous studies have reported an increase in herpes zoster cases associated with periods of increased rainfall and humidity [3,20]. One study reported a latitudinal gradient in herpes zoster seasonality, with a strong correlation observed between seasonal ultraviolet radiation levels and herpes zoster incidence [21]. However, epidemiological sources have generally considered herpes zoster to occur throughout the year because it represents the endogenous reactivation of latent varicella-zoster virus [7]. During the 2020–2025 period, a significant linear increasing trend in herpes zoster case rates was observed in our study. However, this increase was not continuous over the years; herpes zoster case rates decreased in 2022 and 2023, followed by an increase in 2024 and 2025, reaching the highest rate in 2025. The decline in visit-adjusted herpes zoster rates observed in 2022 and 2023 may partly reflect post-pandemic changes in healthcare-seeking behavior, with patients becoming more likely to seek outpatient care for relatively minor complaints [14]. The resulting relative increase in the overall number of dermatology outpatient visits may have increased the denominator, thereby contributing to the lower herpes zoster case rates observed during these years. In our study, the highest herpes zoster case rate was observed in 2025, consistent with the reported global trend toward increasing herpes zoster incidence, which may be partly attributable to population aging and the widespread use of immunosuppressive therapies [6,22]. It is estimated that at least 20–30% of the population and up to 50% of individuals who reach the age of 85 will be affected by herpes zoster [23]. According to a systematic review, most studies that evaluated temporal trends reported an increase in herpes zoster incidence over time [24]. According to data from the Global Burden of Disease database, herpes zoster-associated mortality among individuals aged >65 years ranges from 0.0022 to 82.21 deaths per 100,000 population [4]. Herpes zoster can usually be diagnosed clinically based on the characteristic appearance of the rash. In atypical cases of herpes zoster, laboratory confirmation may be obtained by polymerase chain reaction (PCR), direct immunofluorescence assay, viral culture, or histopathological examination of a skin biopsy (Figure 7 and Figure 8). PCR detection of varicella-zoster virus DNA in vesicular fluid is considered the most sensitive and specific method for confirming herpes zoster. PCR testing can be performed on specimens obtained from skin lesions, blood, plasma, cerebrospinal fluid, and bronchoalveolar lavage fluid [8]. Postherpetic neuralgia is the most common complication of herpes zoster and may substantially impair quality of life, with an impact comparable to that associated with congestive heart failure, depression, acute myocardial infarction, and diabetes mellitus [15]. Antiviral-resistant VZV strains may emerge in immunocompromised patients, particularly during prolonged antiherpesvirus therapy [25]. Herpes zoster vaccination is an effective preventive strategy for reducing the risk of VZV reactivation and antiviral-resistant VZV infections [17,25]. Two vaccines have been developed for the prevention of herpes zoster: the zoster vaccine live (ZVL), licensed in 2006, and the recombinant zoster vaccine (RZV), introduced in 2017 [1]. Individuals with herpes zoster can transmit VZV to susceptible seronegative contacts, resulting in varicella rather than herpes zoster. Childhood varicella vaccination may influence the epidemiology of herpes zoster in two distinct ways. First, as vaccinated children reach adulthood, fewer individuals will harbor latent wild-type VZV, potentially reducing the incidence of herpes zoster. Second, in the short term, reduced exposure to children with varicella may decrease exogenous boosting of VZV-specific immunity in adults with latent VZV. As exogenous boosting is thought to protect against VZV reactivation, its reduction may temporarily increase the incidence of herpes zoster and lower the age at disease onset. Mathematical models suggest that the increase in herpes zoster incidence may persist for more than 30 years [26]. The largest proportion of herpes zoster cases in our study was observed in the 60–69-year age group (21.8%). A population-based study similarly reported the highest herpes zoster incidence in the same age group (22.4 per 1000 person-years) [15]. A population-based study spanning 60 years reported a more than fourfold increase in herpes zoster incidence across all age groups and both sexes. However, the rate of increase did not differ significantly before and after the introduction of varicella vaccination [16]. In our country, a universal single-dose varicella vaccination program for children aged 12 months was introduced in 2013, although a two-dose schedule is recommended for licensed live attenuated varicella vaccines. The recombinant zoster vaccine is available in our country but is not currently included in the national immunization program [27]. Herpes zoster surveillance has been recommended to assess the potential impact of varicella vaccination on herpes zoster incidence [5]. Understanding seasonal patterns and long-term trends in herpes zoster occurrence is important for informing public health planning, optimizing healthcare resource allocation, and guiding preventive strategies [10]. In our study, no significant seasonal variation in herpes zoster case rates was observed. In contrast to our findings, several studies have demonstrated significant seasonal variation, most commonly characterized by a peak during the summer months [9,10,18]. These discrepancies may reflect differences in geographic location, climatic conditions, study populations, and methodological approaches used to assess seasonality. Several population-based studies have also reported no significant seasonal variation in herpes zoster occurrence, with the earliest published in 1957 and the most recent in 2025 [17,28]. In accordance with these observations, the Centers for Disease Control and Prevention (CDC) Pink Book indicates that herpes zoster does not exhibit a distinct seasonal pattern and occurs throughout the year [29]. In this study, no statistically significant monthly variation in herpes zoster case rates was detected, even after adjusting for calendar year and variations in outpatient attendance using negative binomial regression. This finding suggests that the absence of a significant monthly pattern was not solely attributable to differences in outpatient visit numbers or annual variations in case rates. The discrepancies among studies investigating herpes zoster seasonality may partly reflect differences in study design, data presentation, and statistical methods. For example, Kim et al. [15] reported no seasonal variation based on the monthly and seasonal distributions of herpes zoster cases during a single year, whereas Miyachi et al. [10] identified a summer peak using seasonal-trend decomposition of nationwide Japanese health insurance claims data. Interestingly, two population-based studies from South Korea reported contrasting findings: Kim et al. [15] found no significant monthly variation in herpes zoster occurrence using data from a single year (2011), whereas Kwon et al. [22] identified a summer peak in a seven-year nationwide analysis (2010–2016), suggesting that differences in observation periods, sample sizes, and analytical approaches may contribute to the inconsistent findings. Diehl et al. [19] reported summer and winter peaks based on monthly and cumulative daily incidence patterns over 14 years. These differences suggest that the duration of observation, temporal resolution of the data, and statistical approaches may influence the identification of seasonal patterns. Furthermore, studies have used different epidemiological measures, including absolute case counts, population-based incidence rates, and healthcare visit-adjusted rates, which may limit direct comparisons. In this study, herpes zoster case rates were calculated per 1000 dermatology outpatient visits, and negative binomial regression revealed no statistically significant monthly variation after adjustment for calendar year and outpatient attendance. Nevertheless, visit-adjusted case rates are not directly equivalent to population-based incidence rates, and differences in healthcare utilization may influence the observed patterns. Beyond methodological differences, geographical location, climatic conditions, and population characteristics may contribute to the inconsistent findings. Bakker et al. [21] reported geographical differences in herpes zoster seasonality associated with latitude and seasonal ultraviolet radiation, while Rahman et al. [3] and Adinda et al. [20] described associations with rainfall and humidity. In contrast, Ochal et al. [17] found no clear seasonal pattern but reported an association between herpes zoster occurrence and strong heat stress. These findings suggest that environmental factors may influence herpes zoster occurrence without necessarily producing consistent seasonal patterns across geographical regions. Differences in population age structure, immunosuppression, vaccination practices, and healthcare-seeking behavior may also contribute to the heterogeneous findings. Therefore, the discrepancies among studies are likely multifactorial, highlighting the need for larger multicenter studies employing standardized epidemiological measures and comparable statistical approaches.

This study has several limitations, including its single-center, retrospective design, the restriction of the study period to nine years following the hospital’s transition from a military hospital to a public hospital in October 2016, and the lack of information on patients’ vaccination and immunosuppression status. Furthermore, changes in healthcare utilization during the COVID-19 pandemic may have influenced the observed visit-adjusted herpes zoster case rates. Despite these limitations, findings from different geographical and healthcare settings contribute to a broader understanding of herpes zoster epidemiology and inform preventive strategies. More conclusive evidence may be obtained from large-scale, multicountry studies conducted concurrently across diverse populations.

## 5. Conclusions

Varicella-zoster virus causes varicella during primary infection and herpes zoster following the reactivation of latent virus [1]. Herpes zoster may lead to severe complications, resulting in increased hospitalization rates [5]. Epidemiological sources have generally considered herpes zoster to occur throughout the year because it represents the endogenous reactivation of latent varicella-zoster virus [7]. More recent studies on large populations have demonstrated significant seasonal variation with a peak during the summer months [9,10]. In our study, no significant monthly and seasonal variation in herpes zoster case rates was observed. Several population-based studies have also reported no significant seasonal variation in herpes zoster occurrence, with the earliest published in 1957 and the most recent in 2025 [17,28]. In accordance with these observations, the CDC Pink Book indicates that herpes zoster does not exhibit a distinct seasonal pattern and occurs throughout the year [29]. No statistically significant seasonal variation in herpes zoster occurrence was detected in this cohort, supporting the importance of maintaining year-round preventive strategies, including vaccination.

## Figures and Tables

**Table 1:** Demographic characteristics of herpes zoster cases.

	*n* = 1493
Gender	
Female	784 (52.5%)
Male	709 (47.5%)
Mean ± SD (standard deviation)	53.4 ± 19.0
Age (years), median (25th–75th percentile)	56 (41–67)
Age Group (years)	
1–17	52 (3.5%)
18–29	168 (11.3%)
30–39	123 (8.2%)
40–49	203 (13.6%)
50–59	318 (21.3%)
60–69	325 (21.8%)
70–79	210 (14.1%)
≥80	94 (6.3%)

**Table 2:** Numbers of herpes zoster cases and total outpatient visits by year, month, and season.

		Herpes Zoster Cases, *n*	Total Outpatient Visits, *n*
Year	2017	116	33,924
	2018	219	50,540
	2019	187	49,474
	2020	122	31,238
	2021	111	27,066
	2022	108	39,775
	2023	128	46,280
	2024	218	50,940
	2025	284	54,562
Month	January	124	30,251
	February	117	33,390
	March	120	34,024
	April	115	26,692
	May	111	30,229
	June	104	28,766
	July	101	29,786
	August	122	28,478
	September	127	32,242
	October	157	38,789
	November	157	35,465
	December	138	35,687
Season	Spring	346	90,945
	Summer	327	87,030
	Autumn	441	106,496
	Winter	379	99,328

**Table 3:** Seasonal and annual variation in herpes zoster case rates per 1000 outpatient visits.

Season	2017	2018	2019	2020	2021	2022	2023	2024	2025
Winter	1.59	4.28	4.28	3.34	4.49	3.40	2.90	3.95	5.74
Spring	2.40	4.20	3.13	6.18	4.06	2.40	2.30	4.24	5.86
Summer	4.18	5.11	4.70	2.98	4.32	2.30	2.50	3.01	4.42
Autumn	5.38	3.86	3.41	3.90	3.63	2.72	3.23	5.96	4.99

**Table 4:** Monthly and annual variation in herpes zoster case rates per 1000 outpatient visits.

Month	2017	2018	2019	2020	2021	2022	2023	2024	2025	Monthly Pooled Rate
January	1.90	5.37	5.13	3.45	6.31	3.56	1.65	4.18	5.86	4.10
February	0.51	3.40	5.51	3.11	7.71	3.07	3.20	2.61	6.38	3.50
March	0.59	3.85	2.97	5.81	2.52	2.09	2.42	4.60	6.89	3.53
April	3.47	4.16	4.21	9.04	8.48	2.11	1.63	3.33	7.50	4.31
May	4.28	4.52	2.25	5.34	1.72	3.26	2.72	4.57	4.12	3.67
June	4.15	4.97	5.32	3.18	3.56	2.45	1.29	2.97	4.45	3.62
July	4.72	3.85	4.23	1.07	4.80	2.16	2.57	3.42	3.59	3.39
August	3.69	7.25	4.79	4.98	5.09	2.25	3.53	2.57	5.21	4.28
September	7.08	4.32	4.83	2.40	4.71	1.56	2.20	4.19	4.98	3.94
October	5.82	4.51	1.67	4.17	3.86	3.19	3.11	6.58	4.43	4.05
November	4.11	2.91	4.59	5.60	2.51	3.50	4.21	7.49	5.57	4.43
December	2.66	4.25	3.01	3.80	2.13	3.61	3.55	5.29	5.31	3.87
Annual Pooled Rate	3.42	4.33	3.78	3.91	4.10	2.72	2.77	4.28	5.21	

**Table 5:** Comparison of visit-adjusted herpes zoster (hz) case rates across months.

Month	HZ Cases, *n*	Total Outpatient Visits, *n*	Cases per 1000 Visits	*p* *
January	124	30,251	4.10	0.453
February	117	33,390	3.50	
March	120	34,024	3.53	
April	115	26,692	4.31	
May	111	30,229	3.67	
June	104	28,766	3.62	
July	101	29,786	3.39	
August	122	28,478	4.28	
September	127	32,242	3.94	
October	157	38,789	4.05	
November	157	35,465	4.43	
December	138	35,687	3.87	

* Chi-square test.

**Table 6:** Comparison of visit-adjusted herpes zoster case rates across years.

Year	HZ Cases, *n*	Total Outpatient Visits, *n*	Cases per 1000 Visits	Years with Significant Pairwise Differences
2017	116	33,924	3.42	2025
2018	219	50,540	4.33	2022, 2023, 2025
2019	187	49,474	3.78	2025
2020	122	31,238	3.91	–
2021	111	27,066	4.10	–
2022	108	39,775	2.72	2018, 2024, 2025
2023	128	46,280	2.77	2018, 2024, 2025
2024	218	50,940	4.28	2022, 2023
2025	284	54,562	5.21	2017, 2018, 2019, 2022, 2023

Overall comparison: χ^2^ = 60.576, df = 8, *p* < 0.001. Linear-by-linear association (2017–2025): χ^2^ = 3.842, df = 1, *p* = 0.051. Linear-by-linear association (2020–2025): χ^2^ = 14.431, df = 1, *p* < 0.001. The 2020–2025 trend analysis was performed as a post hoc exploratory analysis.

**Table 7:** Comparison of visit-adjusted herpes zoster case rates across seasons.

	Cases per 1000 Outpatient Visits (Number of Cases/Number of Outpatient Visits)	*p* *
Winter	3.82 (379/99,328)	0.486
Spring	3.80 (346/90,945)	
Summer	3.76 (327/87,030)	
Autumn	4.14 (441/106,496)	

* Chi-square test.

## Data Availability

The data in this study are available upon request from the corresponding author due to privacy restrictions.

## Abbreviations

The following abbreviations are used in this manuscript:

CDC	Centers for Disease Control and Prevention
df	Degrees of freedom
H&E	Hematoxylin and Eosin
HHV-3	Human herpesvirus 3
HZ	Herpes zoster
ICD-10	International Classification of Diseases, 10th Revision
PCR	Polymerase chain reaction
RZV	Recombinant zoster vaccine
SD	Standard deviation
VZV	Varicella-zoster virus
ZVL	Zoster vaccine live
